# High photon-phonon pair generation rate in a two-dimensional optomechanical crystal

**DOI:** 10.1038/s41467-025-57948-7

**Published:** 2025-03-15

**Authors:** Felix M. Mayor, Sultan Malik, André G. Primo, Samuel Gyger, Wentao Jiang, Thiago P. M. Alegre, Amir H. Safavi-Naeini

**Affiliations:** 1 Department of Applied Physics and Ginzton Laboratory, Stanford University, Stanford, CA USA; 2 Instituto de Física Gleb Wataghin, Universidade Estadual de Campinas (UNICAMP), Campinas, SP Brazil

**Keywords:** Photonic crystals, Single photons and quantum effects, Quantum optics

## Abstract

Integrated optomechanical systems are a leading platform for manipulating, sensing, and distributing quantum information, but are limited by residual optical heating. Here, we demonstrate a two-dimensional optomechanical crystal (OMC) geometry with increased thermal anchoring and a mechanical mode at 7.4 GHz, well aligned with the operation range of cryogenic microwave hardware and piezoelectric transducers. The eight times better thermalization than current one-dimensional OMCs, large optomechanical coupling rates, *g*_0_/2*π*  ≈  880 kHz, and high optical quality factors, *Q*_opt_ = 2.4 × 10^5^, allow ground-state cooling (*n*_m_ = 0.32) of the acoustic mode from 3 K and entering the optomechanical strong-coupling regime. In pulsed sideband asymmetry measurements, we show ground-state operation (*n*_m_ < 0.45) at temperatures below 10 mK, with repetition rates up to 3 MHz, generating photon-phonon pairs at  ≈ 147 kHz. Our results extend optomechanical system capabilities and establish a robust foundation for future microwave-to-optical transducers with entanglement rates exceeding state-of-the-art superconducting qubit decoherence rates.

## Abstract (web summary)

Here the authors overcome the temperature increase in integrated optomechanical systems, harnessing two-dimensional optomechanical crystal geometry. These results set the ground for microwave-to-optical transducers with entanglement rates overcoming the decoherence rates of state-of-the-art superconducting qubits.

## Introduction

The integration of mechanical systems with photonic circuits^1^ offers a versatile platform for high-performance sensors^2^, signal processing^3^ and exploring macroscopic systems in the quantum regime^4^. These optomechanical devices^5^ allow for precise detection of motion using light at and beyond the standard quantum limit^6^, enabling the measurement of acceleration^7^, displacement^8^, mass^9^, forces^10^ at both room and cryogenic temperatures, and the transduction of quantum information between disparate energy scales^11,12^.

Silicon-based optomechanical crystals (OMC)^13^ are particularly promising systems with exceptional photon-phonon cooperativity. This is due to their high optical and mechanical quality factors combined with large optomechanical couplings, enabled by their sub-wavelength modal volumes. Their operation at the quantum level is possible using either milli–Kelvin cryogenic environments or by laser cooling the mechanical modes into their ground state from precooled conditions^14^. This enables the preparation of mechanical quantum states^15^, optical squeezed states^16^, as well as the demonstration of entanglement between mechanical resonators^17^. The availability of high-frequency mechanical modes in the GHz regime, combined with piezoelectric^18,19^ or electrostatic coupling^20^, make these structures ideal for coherently transducing quantum signals from the microwave to the optical domain to build superconducting qubits-based quantum networks^21^.

OMCs are typically nanobeams patterned along one-dimension (1D)^22^ (≈500 nm in width) that are released from the substrate and co-confine optical and mechanical modes. In such devices, heat only dissipates to the surrounding material along the length of the beam. The residual heating through linear and nonlinear absorption^23,24^, leads to resonance shifts and optical instabilities at room temperature^25^, while at cryogenic temperatures heating of the mechanical mode hinders operation in the quantum limit. Therefore various strategies are being pursued to improve thermalization and minimize residual heating. First, utilizing non-suspended OMCs may help with thermal anchoring^26^ as the heat can propagate into the bulk of the chip. Second, cooling the device using a buffer gas, as demonstrated with ^3^He at  ≈2 K^27^ has been effective at maintaining low temperatures while laser driving. However, immersion in liquid ^4^He, which transitions to a superfluid below 2.17 K, introduces new and interesting superfluid-structure interactions and dynamics which seem to complicate operating the device as a transducer^28,29^.

One approach to reducing the challenges posed by heating is to develop two-dimensional (2D) OMCs^30–35^. The initial demonstrations of these devices had limited coupling rates and a complicated mechanical mode spectrum, which largely negated their purported benefits in thermalization. A major advance in 2D OMC design^31^ led to photon-phonon coupling rates on par with their 1D counterparts while demonstrating improved thermalization in continuous-wave (CW) operation. Nonetheless, 2D OMCs are yet to be incorporated into optomechanical transducers. Part of the challenge is due to their generally higher microwave operating frequency – more than 10 GHz vs. less than 5 GHz for 1D OMCs. This is because fully connected 2D structures are generally stiffer than 1D devices. Higher operation frequency makes piezoelectric coupling to the mode more difficult due to fabrication limiting minimum dimensions and increasing the density of states, leading to a growth in spurious mechanical modes that reduce robustness and create additional loss channels in the transducer. Moreover, it requires a higher optical driving power due to a larger sideband detuning. Secondly, integrated optomechanical transducers have mostly operated as optically pulsed devices to avoid issues related to heating dynamics which kick in at a slower time scale than the pulse duration. It is therefore important to demonstrate the advantages of the 2D OMCs while operating them in optically pulsed mode.

Here, we present a novel Si-based two-dimensional OMC with high optical and mechanical quality factors. In contrast to prior demonstrations, the device operates at lower frequency, which allows for straightforward integration with a piezoelectric transducer^18,36,37^, reduces heating through lower required pump power, and conveniently operates in the superconducting qubit frequency band. The enhanced thermalization allows us to demonstrate ground-state sideband cooling by CW optical driving starting from a fridge temperature of  ≈3 K. Moreover, due to improved power handling, the device operates in a stable manner at sufficiently high power to achieve continuous strong coupling between the optical and mechanical modes – a first for silicon optomechanical devices to our knowledge. We demonstrate pulsed operation at  <10 mK at repetition rates as large as 3 MHz with the mechanical mode occupation still in the ground state. Our findings provide a pathway to high-repetition entanglement creation utilizing optomechanical transducers^38–40^.

## Results

### Two-dimensional C-band optomechanical crystal

**Fig. 1:**
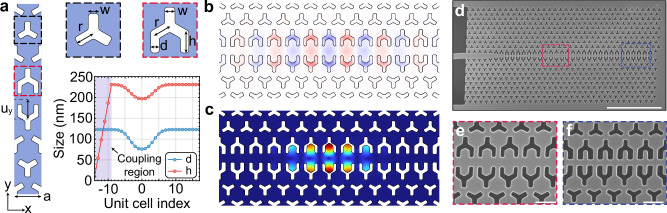
2D Optomechanical crystal design and implementation. **a** Schematic of the waveguide unit cell and definition of parameters. Typical parameters are *a* = 448 nm, *w* = 93 nm, *r* = 172 nm, and a fillet radius of 25 nm for a silicon thickness of 220 nm. The dimensions *d* and *h* are modified along the length of the waveguide to confine the optical and acoustic fields. To couple light into the cavity, *h* is linearly tapered down. **b**, **c** FEM simulations of the full cavity displaying the electric field *E*_*y*_ of the fundamental optical mode and the displacement profile of the mechanical mode of interest. **d** Scanning electron micrograph of the fabricated optomechanical crystal. Scale bar is 5 μm. **e**, **f** Close-up of the defect and mirror regions of the waveguide. Scale bars are 500 nm.

Our Si-based two-dimensional OMC is built on a hybrid b-dagger (boomerang-dagger) design, where the optomechanical shield is based on the boomerang unit cell (identical to the blade cell proposed in ref. ^41^) and the defect region is designed in a dagger shape. Figure 1a depicts a schematic of the effective waveguide unit cell of the device together with the parameters defining our design. We align the *x*-axis of our device along the [1, 0, 0] crystal orientation of silicon, where the stiffness coefficients are lower resulting in lower mechanical frequencies^42^. Optomechanical confinement is achieved by adiabatically modifying the dagger parameters *d* and *h* as shown in Fig. 1a such as to locally create a density of states inside the photonic and phononic bandgaps (see Supplementary Fig. 2). We use finite element method (FEM) simulations combined with particle swarm optimization to simultaneously optimize the optical and mechanical quality factors, *Q*_opt_ and *Q*_m_, and the single-photon optomechanical coupling rate *g*_0_ while minimizing the mechanical frequency *ω*_m_. The mode profiles of interest are displayed in Fig. 1b and c, showing wavelength-scale confinement in both the optical and acoustic domains. Our devices operate at an optical frequency *ω*_c_/2* π *≈ 193 THz, within the telecom C-band, at *ω*_m_/2*π* ≈ 7.5 GHz, with radiation-limited *Q*_opt_ > 5 × 10^6^ and *Q*_m_ > 1 × 10^9^. We compute the optimal *g*_0_ accounting for both moving boundary and photoelastic contributions^43^, yielding *g*_0_/2*π* ≈ 950 kHz. The fabricated device (see “Methods” section) is shown in the scanning electron micrograph in Fig. 1d with a close-up of the center defect and mirror in Fig. 1e and f, respectively.

**Fig. 2:**
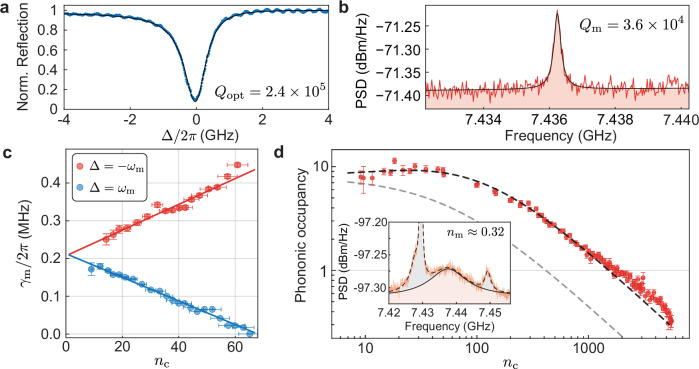
Ground-state cooling of the mechanical mode. **a** Room-temperature optical spectrum as a function of the laser-cavity detuning Δ = *ω*_l_ − *ω*_c_. The fit (black) takes into account the Fano lineshape of the mode. **b** Mechanical response of the OMC at *T* = 3 K. **c** Backaction cooling and amplification as a function of photon number (*n*_c_), under both red- and blue-detuned excitation. **d** Ground-state cooling of the mechanical mode. The black (gray) dashed curve depicts a cooling model including (neglecting) parasitic optical heating. Inset: mechanical spectrum for the highest intracavity occupation in the experiment, *n*_c_ ≈ 5500. Dashed line: multimode fit of the mechanical spectrum; solid line: fit results for the mode of interest. The error bars represent one standard deviation of the parameter estimated through uncertainty propagation accounting for both nonlinear fit residuals and experimental uncertainties.

We study the devices at room temperature, 3 K, and 10 mK. The cryogenic measurements were done at the mixing chamber plate of a dilution refrigerator before and after condensation. We measure the room-temperature optical response using a lensed fiber to send and collect light from an on-chip waveguide butt-coupled to the b-dagger OMC. A typical reflection spectrum is shown in Fig. 2a displaying the fundamental optical mode at *ω*_c_/2*π* = 191.7 THz (1563.5 nm) as a function of the laser-cavity detuning Δ = *ω*_l_ − *ω*_c_. A Lorentzian fit to the data yields a loaded *Q*_opt_ = 2.4 × 10^5^ (2.1 × 10^5^ at 3 K), with an extrinsic coupling rate *κ*_e_/2*π* ≈ 290 MHz (measured through coherent sideband spectroscopy and reflection spectrum), putting the device close to critical coupling. The mechanical spectrum at *T* = 3 K is assessed through amplitude fluctuations imparted on the reflected optical signal by the thermo-mechanical motion of the OMC. This signal is then measured using a high-speed photodetector and a real-time spectrum analyzer yielding the photocurrent power spectral density (PSD). The mechanical breathing mode of the OMC is found at *ω*_m_/2*π* ≈ 7.436 GHz with *Q*_m_ = 3.6 × 10^4^ as shown in Fig. 2b.

Our device operates well within the sideband-resolved regime *ω*_m_ > *κ* (*κ*/2*π* ≈ 800 MHz). In this regime, the linearized optomechanical interaction under a blue-detuned, Δ = *ω*_m_ (red-detuned, Δ = −*ω*_m_), coherent excitation is reduced to a two-mode squeezing (beam splitter) interaction, with Hamiltonian $\hat{H}=\hslash g(\hat{a}\hat{b}+{\hat{a}}^{{{\dagger}} }{\hat{b}}^{{{\dagger}} })$ ($\hat{H}=\hslash g(\hat{a}{\hat{b}}^{{{\dagger}} }+{\hat{a}}^{{{\dagger}} }\hat{b})$)^5^. This interaction leads to the mechanical mode’s effective amplification (cooling), associated with a narrowing (broadening) of its linewidth. Here, $g={g}_{0}\sqrt{{n}_{{{{\rm{c}}}}}}$ is the linearized effective optomechanical coupling, enhanced by the number of photons circulating in the cavity, *n*_c_, and $\hat{a},\hat{b}$ denote the annihilation operators for photons and phonons, respectively.

In Fig. 2c we characterize the mechanical linewidth, *γ*_m_, as a function of *n*_c_, for both red- (Δ < 0) and blue-detuned (Δ > 0) excitation. In the sideband-resolved regime, ${\gamma }_{{{{\rm{m}}}}}\approx {\gamma }_{{{{\rm{m}}}}}^{0}(1\pm C)$, where $C=4{g}_{0}^{2}{n}_{{{{\rm{c}}}}}/(\kappa {\gamma }_{{{{\rm{m}}}}}^{0})$ is the optomechanical cooperativity and ${\gamma }_{{{{\rm{m}}}}}^{0}$ is the bare mechanical linewidth. A linear fit to the measured data yields *g*_0_/2*π* = 920(30) kHz (Δ = −*ω*_m_) and *g*_0_/2*π* = 880(30) kHz (Δ = *ω*_m_), in good agreement with simulation results.

### Ground-state cooling of the mechanical mode

A mechanical resonator placed at a temperature *T* ≪ *ℏω*/*k*_B_, will occupy its ground state nearly perfectly. For a 7 GHz resonator this condition is achieved at the 10 mK base temperature of dilution refrigerators. Operating at such low temperatures poses many challenges largely rooted in the extremely small (microwatt-level) available cooling powers, vanishing heat capacity, and small thermal couplings. It is therefore important to consider whether quantum operation is possible at higher temperatures where less exotic cryogenic systems provide orders of magnitude greater cooling power, making scaling to more transducers and control lines more realistic. Higher temperature has been previously considered as a route to improved microwave-to-optical transducers^27,39,44^. The first demonstration of optical ground-state cooling started from a temperature of roughly *T*_ini_ = 20 K^14^. Rather counter-intuitively, for silicon optomechanical devices, laser cooling from lower starting temperatures becomes more challenging, since the temperature increase due to optical absorption heating grows more quickly as the temperature is reduced than the advantage of starting with a lower initial phonon population^45^. Operation in the quantum regime from a lower starting temperature than 20 K is however important for developing transducers, since the superconducting materials used in such devices will need to have a critical temperature *T*_c_ ≫ *T*_ini_ to avoid excess quasiparticle losses, and for many common materials *T*_c_’s are in the range of 10–15 K.

Starting from a base temperature of 3 K, we laser cool our 2D OMC device into its quantum ground state. In Fig. 2d, we show the inferred mechanical mode occupation as a function of the laser-driven intracavity photon number *n*_c_. The phonon occupancy, *n*_m_, is obtained through the area under the measured thermo-mechanical response of the acoustic mode at 7.436 GHz. In this measurement, we calibrated the photodetection gain for different input powers by intensity modulating the laser at 7.446 GHz and measuring its PSD. The input laser is filtered to mitigate contributions from technical laser noise in the measured mechanical spectrum^46^, reducing the maximum excitation power attainable in the experiment. At low optical powers (*n*_c_ < 10), we assume the acoustic mode is thermalized to the plate temperature of 3 K, with a phononic occupancy *n*_m_ = *n*_th_ ≈ 7.95. At high input powers, *n*_c_ ≈ 5500, the effective temperature of the mechanical mode is cooled to *T*_eff_ ≈ 250 mK, yielding *n*_m_ ≈ 0.32 ± 0.04, corresponding to a 75% probability of ground-state occupation. The black dashed line depicts the theoretical prediction for cooling in the presence of a phenomenological increase in the acoustic thermal bath temperature with *n*_c_ (see “Methods” section). In the high intracavity field regime, several modes appear in the mechanical spectra and are included in our fits (see inset of Fig. 2d) for an accurate calibration of *n*_m_.

### Optomechanical strong coupling

Another straightforward consequence of the better thermal anchoring of two-dimensional optomechanical crystals is the strong suppression of thermo-optic nonlinearities. The temperature increase in typical devices red-shifts the optical resonance, leading to an unstable optical response for a sufficiently strong red-detuned pump. For 1D OMCs, this effect usually constrains *n*_c_ < 2000^14,47,48^ precluding the onset of the strong-coupling regime (4*g* > *κ*)^49^, where the optical and mechanical modes hybridize, hence enabling the coherent state swap between photonic and phononic domains.

**Fig. 3:**
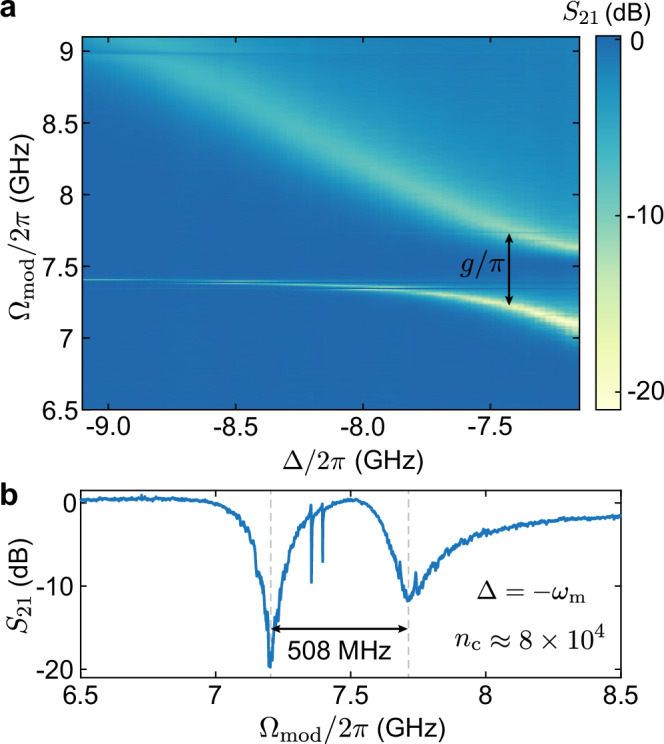
Optomechanical strong coupling. **a** Measurement of optomechanically induced transparency (OMIT) as a function of laser detuning Δ. The avoided crossing at ${\Omega }_{{{{\rm{mod}}}}}\approx -{\omega }_{{{{\rm{m}}}}}$ results from the strong coupling between the optical and mechanical mode of the OMC. **b** Line-cut of (**a**) at Δ = −*ω*_m_. From the splitting, we infer that we are in the strong-coupling regime (*g*/*π* = 508 MHz > *κ*/4*π*). The strong pump field makes additional weakly coupled mechanical modes visible.

To observe strong coupling, we perform optomechanically induced transparency measurements. We intensity-modulate a pump laser detuned by Δ with respect to the optical mode of the OMC at a frequency ${\Omega }_{{{{\rm{mod}}}}}$ using an electro-optical modulator and a vector network analyzer. The latter is used to measure the coherent response of the b-dagger OMC, ${S}_{21}({\Omega }_{{{{\rm{mod}}}}})$. When ${\Omega }_{{{{\rm{mod}}}}}={\omega }_{{{{\rm{m}}}}}$, the anti-Stokes sideband arising from the modulation interferes with mechanically scattered photons giving rise to a transparency window in ∣*S*_21_∣, indicating a hybridization of mechanical and optical loss channels. We measure this response as a function of the pump detuning Δ (Fig. 3a) for an on-chip pump power of 9.4 m W (corresponding to *n*_c_ ≈ 8 × 10^4^). When the pump is red-detuned by Δ = −*ω*_m_, we observe an avoided crossing with a frequency splitting *g*/*π* = 508 MHz (Fig. 3b). From a fit of *S*_21_ at large detuning Δ ≈ −9.5 GHz, we obtain an optical linewidth *κ*/2*π* = 870 MHz (placing the cooperativity at *C* ≈ 1400). This puts our system into the strong-coupling regime (4*g*/*κ* ≈ 1.17), so far elusive in wavelength-scale silicon devices.

### Pulsed sideband thermometry at *T* ≲ 10 mK

**Fig. 4:**
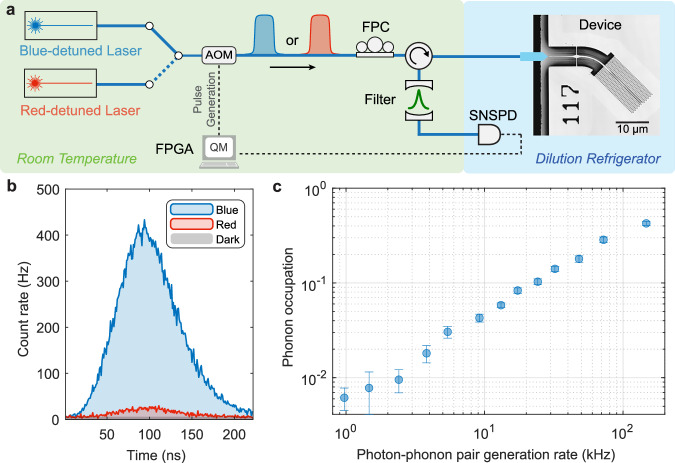
Pulsed sideband asymmetry measurement. **a** Simplified setup of the pulsed sideband asymmetry measurement with the device at *T* ≲ 10 mK. Blue- or red-detuned optical pump pulses of length *τ* = 80 ns incident on the device generate sideband photons on-resonance with the optical cavity. The reflected light, containing residual pump and sideband photons, is filtered to suppress the pump. The transmitted sideband photons are detected with a single photon detector, and the photon clicks are time-tagged. **b** Time trace of the sideband photons generated using blue- and red-detuned pump pulses, averaged over 1.8 billion experimental runs, for a scattering probability of  ≈5% and a pulse repetition rate of 188 kHz corresponding to a photon-phonon pair generation rate of  ≈9.2 kHz. From the asymmetry between the blue and red count rates, we extract a phonon occupation of  ≈0.043. **c** Phonon occupation for various photon-phonon pair generation rates (varying pulse repetition rates with the scattering probability fixed at  ≈5%) quantifying the effect of optically induced heating of the mechanical mode. The error bars represent the standard error over multiple repeated sets of measurements (ranging from 24 to 2079 for different data points) with the nominal phonon occupation values averaged over total experimental runs ranging from 0.2 to 18.7 billion.

We characterize a second device at *T* ≲ 10 mK using pulsed sideband asymmetry measurements on the mechanical breathing mode. Figure 4a shows a simplified measurement setup. Starting with a blue- and a red-detuned laser, each locked to a Fabry–Pérot filter that is parked one mechanical frequency away from the optical cavity resonance, we use two acousto-optic modulators (AOM) in series to generate optical pulses of length *τ* = 80 ns with an on-chip peak pump power *P*_peak_ ≈ 7.4 μW (*n*_c_ ≈ 33.8). Through optomechanical interaction, a fraction of the optical pump photons get scattered (or frequency-shifted) into sideband photons resonant with the optical cavity. The reflected light, consisting of residual pump and sideband photons, then passes through a pair of narrowband Fabry–Pérot cavities (bandwidth  ≈15 MHz) that suppress the pump photons with a joint suppression of  >90 dB with respect to the sideband photons. The transmitted sideband photons are then detected by a superconducting nanowire single-photon detector (SNSPD), and the recorded photon clicks are time-tagged. The photon clicks are averaged over multiple trials of the experiment, interleaved every 20 min between the blue- and red-detuned laser. Pulse generation and data acquisition are performed using an FPGA-based real-time controller.

Using the optical pulse parameters and the measured device properties, we estimate the photon-phonon Stokes scattering probability ${p}_{{{{\rm{s}}}}}\approx C{\Gamma }_{{{{\rm{m}}}}}\tau \approx 4{g}_{0}^{2}{n}_{{{{\rm{c}}}}}\tau /\kappa \approx 5\%$, calculated assuming the mechanical mode in the ground-state. The photon-phonon pair generation rate, given by the experiment repetition rate times the scattering probability (*R*_rep_*p*_s_), is a direct measure of the ability to generate entanglement using future OMC-based transducers. Figure 4b shows a time-resolved histogram of the optomechanically scattered sideband photons averaged over multiple experimental runs for blue- and red-detuned lasers for an optical pulse repetition rate of 188 kHz. This corresponds to a photon-phonon pair generation rate of  ≈9.2 kHz. Dark counts arising from spurious light and detector noise are shown in gray (≈5 Hz). The asymmetry between the sideband count rates under blue-detuned operation (proportional to *p*_s_(*n*_m_ + 1)) and red-detuned operation (proportional to *p*_s_*n*_m_) provides a direct way to quantify the thermal occupation of our mechanical mode^50,51^. The asymmetry in count rates stems from the quantum nature of the processes involved: while transitions to higher phonon states induced by the blue-detuned pump are always possible, phonon annihilation due to the interaction with the red-detuned pump is completely suppressed if the mechanical mode is in the ground state. From the asymmetry measured in Fig. 4b, we extract a phonon occupation *n*_m_ = 0.043 ± 0.004.

The pulsed sideband asymmetry measurements are performed for various photon-phonon pair generation rates (by varying the optical pulse repetition rates and keeping the scattering probability fixed at  ≈5%), and the resulting phonon occupation is shown in Fig. 4c, revealing the effect of optically induced heating of the mechanical mode. While the thermal occupation increases with the increasing repetition rate as expected, our device stays in the ground state for a repetition rate as high as 3.012 MHz, corresponding to a photon-phonon pair generation rate of  ≈147 kHz, with a thermal occupation *n*_m_ = 0.42 ± 0.02. Our device demonstrates one of the lowest reported thermal occupations for an OMC under pulsed operation, representing a reduction by roughly a factor of eight in the thermal occupation of the mechanical mode compared to 1D structures at similar repetition rates and photon-phonon scattering probabilities^52,53^.

## Discussion

In summary, we designed, fabricated, and demonstrated a proof-of-principle experiment for high-rate photon-phonon pair generation without significant residual heating using a silicon-based 2D optomechanical crystal. Our device operates at acoustic frequencies lower than those previously demonstrated in similar structures, enabling operation at reduced driving powers and favoring future integration to piezoelectric elements.

The enhanced thermal anchoring to the substrate of our design enables ground-state cooling of the mechanical mode and entering the optomechanical strong-coupling regime. These demonstrations open the way for full quantum control of integrated optomechanical systems operated at temperatures of *T* ≈ 3 K, routinely reached by Gifford-McMahon cryocoolers, allowing for simplified cryogenic infrastructure for quantum sensing and communication applications. Moreover, the large cooperativity that we achieve (*C* > 1000) enables a new class of experiments where photonic states can be coherently swapped into the mechanical domain and vice versa, leveraging nanomechanical resonators as sources of optical quantum states. Increasing *Q*_m_, which could be achieved by adding more phononic shields to the crystal structure^54^, could also propel the use of our mechanical mode as a sensor and as a quantum memory for light. Lastly, our optical quality factors are fabrication limited and can exceed a few million in similar two-dimensional photonic structures^55,56^. An enhancement in *Q*_opt_ would reduce the intracavity field required to achieve moderate scattering probabilities and thus further decrease the thermal phononic occupancy under pulsed excitation.

In a next step, our device can be co-integrated with piezoelectric materials to complete a high-rate conversion chain from microwave to optical photons^57^. Scaling up quantum networks for fault-tolerant distributed quantum computing will require transducers capable of stabilizing entanglement between several nodes. For that, the entanglement rates must exceed the decoherence rate of local qubits, while maintaining low added thermal noise^58^. The demonstrated photon-phonon pair generation rates greater than 100 kHz, together with expected microwave (70%) and optical efficiencies (10%), are enough to exceed the ${T}_{2}^{*}=1.5\,{{{\rm{ms}}}}$^59^ (*T*_2_ ≈ 34 ms^60^) of state-of-the-art superconducting qubits (memories), although at an added noise of 10% (1%). Further advances in microfabrication techniques and device design could further improve the thermalization of our system. Allied to the continuous increase in qubit coherence times, this provides a way forward for reliably linking superconducting quantum computing units over long distances.

## Methods

### Fabrication

We start device fabrication by using electron-beam lithography (Raith EBPG 5200+, 100 kV) to pattern the OMCs on a silicon-on-insulator (SOI) chip (220 nm thick silicon device layer, 3 μm thick buried oxide, 725 μm silicon handle, Shin-Etsu,  >3 kΩ ⋅ cm) with AR-P 62.00 e-beam resist and xylenes developer. Next, we do an inductively coupled plasma (ICP) - reactive ion etch (RIE) of the silicon using HBr-based chemistry (Oxford Instruments Plasmalab System 100). The chip is then covered in hexamethyldisilazane (HMDS) followed by 3 μm thick photoresist (MEGAPOSIT SPR220) so that it can be diced to allow optical fiber access to the coupling waveguide. The substrate is subsequently cleaned in a piranha solution (96% sulfuric acid and 30% hydrogen peroxide - 3:1) to remove any organic residue. Finally, the structure is released in 50% hydrofluoric acid (HF) for 2.5 min. Immediately before loading the device into the cryostat, we strip the native oxide with a 2% HF dip.

### Heating model and ground-state cooling

The ground-state cooling data shown in Fig. 2d is fitted using a model accounting for the presence of pump-induced heating through

1 $${n}_{{{{\rm{m}}}}}\left({n}_{{{{\rm{c}}}}}\right)=\frac{1}{1+C}\left({n}_{{{{\rm{th}}}}}^{0}+\frac{{\alpha }_{{{{\rm{sat}}}}}}{1+{\beta }_{{{{\rm{sat}}}}}{n}_{{{{\rm{c}}}}}}{n}_{{{{\rm{c}}}}}\right).$$

We find that introducing a saturable absorption term yields reasonable agreement between the data and the model used to phenomenologically describe the increase in the temperature of the mechanical mode’s thermal bath. The fit results are *α*_sat_ = 0.28, *β*_sat_ = 0.014, indicating an increase in the bath’s temperature to *T* > 10 K (from an initial *T* = 3 K) at *n*_c_ ≈ 5000. We observe a slight deviation between the model and experiment at *n*_c_ > 1000, indicating that further absorption terms would need to be added to correctly describe the experiment at high powers.

### Calibration of the number of photons in the resonator

The number of pumps circulating in the cavity is given by

2 $${n}_{{{{\rm{c}}}}}=\frac{{\kappa }_{{{{\rm{e}}}}}{\dot{N}}_{{{{\rm{in}}}}}}{{\Delta }^{2}+{\kappa }^{2}/4},$$

where ${\dot{N}}_{{{{\rm{in}}}}}$ is the cavity input photon flux.

${\dot{N}}_{{{{\rm{in}}}}}$ is estimated using the fiber to chip coupling efficiency and input power, which is measured using a 95/5 beam splitter and a photodetector before light is sent into the dilution refrigerator (see Supplementary Note 6). The cavity parameters are measured using both coherent sideband spectroscopy and cavity reflection measurements. The discrepancy on the values obtained through different methods is included in the calculation of the uncertainty in *n*_c_.

## Supplementary information

Supplementary Information

Transparent Peer Review file

## Data Availability

The data that support the findings of this study are openly available on Zenodo at 10.5281/zenodo.14722484. Additional data are available from the corresponding author upon reasonable request.
