# Supplementary material for: High photon-phonon pair generation rate in a two-dimensional optomechanical crystal
Source: Nat Commun. 2025 Mar 15;16:2576. doi: 10.1038/s41467-025-57948-7 (PMC11910550; doi:10.1038/s41467-025-57948-7)
Supplement: Supplementary file 1 — Supplementary Information [file 41467_2025_57948_MOESM1_ESM.pdf]

Supplementary information: High photon-phonon pair generation rate in a two-dimensional optomechanical crystal

Felix M. Mayor,[1,*] Sultan Malik,[1,*] André G. Primo,[1,2,*] Samuel Gyger,[1,*] Wentao Jiang,[1] Thiago P. M. Alegre,[2] and Amir H. Safavi-Naeini[1,†]

[1]*Department of Applied Physics and Ginzton Laboratory, Stanford University, 348 Via Pueblo Mall, Stanford, California 94305, USA*

[2]*Instituto de Física Gleb Wataghin, Universidade Estadual de Campinas (UNICAMP), 13083-859 Campinas, SP, Brazil*

(Dated: February 20, 2025)

Supplementary Note 1. MEASURED DEVICE PARAMETERS

The data presented in this work was acquired from two devices from different fabrication runs. Device A (D120) was measured at 3 K after Device B's (D122) thermometry measurements at less than 10 mK were cut short due to infrastructure difficulties.

Supplementary Table I. **Device parameters**

Parameter	Device A (D120)	Device B (D122)	Method
$\omega_o/2\pi$	191.7 THz	193.9 THz	Laser wavelength sweep
$\kappa_o/2\pi$	0.8 GHz	1.1 GHz	Laser wavelength sweep
$\kappa_{o,e}/2\pi$	290 MHz	200 MHz	Laser wavelength sweep and OMIT
$g_o/2\pi$	880 kHz	890 kHz	Mechanical response ($\Delta = \omega_m$)
$\omega_m/2\pi$	7.436 GHz	7.259 GHz	OMIT
$\gamma_m^0/2\pi$	210 kHz	720 kHz	Mechanical response ($\Delta = \omega_m$)
Fiber to chip coupling efficiency	43.0 %	51.1 %	Power meter

Supplementary Note 2. OPTOMECHANICAL CRYSTAL DESIGN

To design the optomechanical shield, we consider multiple factors. It should simultaneously have a large optical bandgap at telecom frequencies and a complete phononic bandgap at frequencies commonly used for superconducting qubits. Ideally, we would also like to have the volume of the unit cell be filled with as much silicon as possible, i.e. we would like a large filling factor. This is to help with thermalization and reduce the number of surfaces that can lead to optical scattering. While the snowflake unit cell [1] fulfills most of these requirements, OMCs using the snowflake have so far been restricted to mechanical frequencies of ~ 10 GHz [2]. This makes it challenging to design and fabricate a transducer that efficiently couples the breathing mode of such an OMC to superconducting circuits. This is mainly due to the smaller required feature sizes of a piezoelectric transducer at higher frequencies to achieve a low acoustic mode density [3]. To address this issue, we use the boomerang unit cell [4] which supports a lower frequency acoustic bandgap than the snowflake at the cost of a smaller relative photonic and phononic bandgap. A schematic of the unit cell is shown in Supplementary Figure 1a and a schematic of the first Brillouin zone in Supplementary Figure 1b. Finite-element method (FEM) simulations with periodic boundary conditions (Supplementary Figure 1c) show that for 220 nm thick silicon and $(a, r, w) = (448\,\text{nm}, 172\,\text{nm}, 93\,\text{nm})$ the optomechanical shield supports an optical pseudo-bandgap for TE-like guided optical waves. This bandgap spans the telecom frequencies $183 - 208$ THz and, importantly, does not close inside the light cone [1]. Additionally, FEM simulations assuming mechanically-isotropic silicon (Supplementary Figure 1d) reveal a 1.30 GHz-wide complete acoustic bandgap centered at 6.99 GHz. The filling factor of the boomerang unit cell is 74.5 %, 15 % larger than the 64.1 % filling factor of the snowflake unit cell.

---

* These authors contributed equally

† safavi@stanford.edu

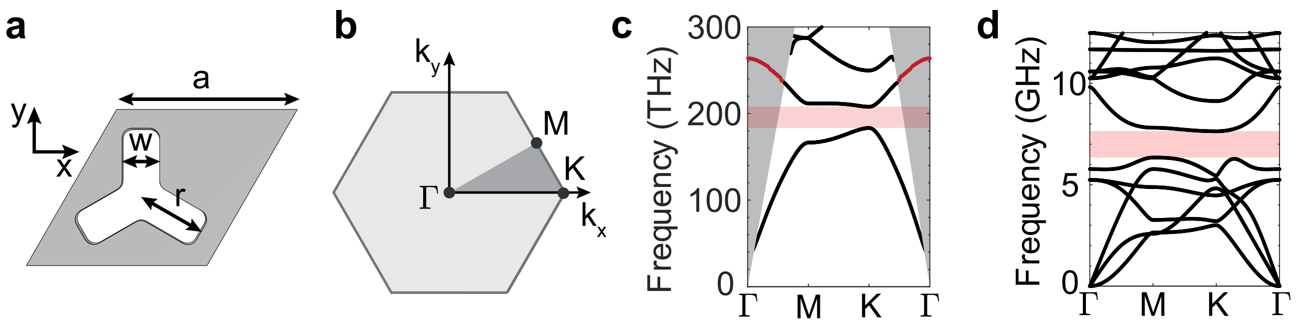

Supplementary Figure 1. **Optomechanical shield design.** **a**, Boomerang unit cell schematic. **b**, First Brillouin zone of the hexagonal lattice with the irreducible zone highlighted. The critical points are also indicated. **c**, Photonic band structure for the even parity modes. The bandgap is highlighted in pink and the light cone in grey. Notice how the bands for the leaky modes (red) inside the light cone do not close the bandgap. **d**, Phononic band structure. The bandgap is highlighted in pink.

As described in the main text, the dagger parameters in each waveguide unit cell are adiabatically transitioned from the center defect (index 0) to the most external cell (index N, for an OMC with $2N+1$ unit cells). The function that parametrizes these variations is given by

$$v_n = v_N - (v_N - v_0)\, 2^{-\left(\frac{n}{\delta_x}\right)^M}, \tag{1}$$

where v_n is a given parameter of the dagger (d or h) for the n-th unit cell. The parameters M and δ_x control how smooth the variation in v is as a function of n and how many waveguide cells will effectively make the transition from v_0 to v_N.

A FEM simulation of the waveguide unit cell shown in Fig. 1a is performed assuming Floquet periodic conditions. It shows the existence of an incomplete (complete) bandgap in the photonic (phononic) domains, as shown in red in Supplementary Figure 2a (b). Importantly, the acoustic bands inside the bandgap display different symmetries than our mode of interest minimizing their coupling, as easily verified through the absence of anti-crossings in the red-shaded area.

Supplementary Figure 2c shows the bandgap – obtained from the same simulations described above – as a function of the unit cell index. Although the mechanical frequency falls outside the bandgap after index 4 in device B, it keeps a high mechanical quality factor in simulations $Q_\mathrm{m} > 5 \times 10^6$, whereas device A has a $Q_\mathrm{m} > 10^9$. In practice, fabrication imperfections allied to the small acoustic bandgaps give rise to a radiation-limited measured Q_m. Adding a surrounding array of rectangular phononic shields could boost our Q_m, at the cost of potentially worse thermal anchoring to the substrate and larger ground state reinitialization times, becoming a less favorable configuration for transduction applications.

All design parameters of Device A and B are provided in Table II.

Supplementary Table II. **Design parameters**

Parameter	Device A (D120)	Device B (D122)
a	448 nm	448 nm
w	92 nm	93 nm
r	167 nm	172 nm
d_0	70 nm	76 nm
h_0	194.5 nm	196.9 nm
d_{17}	122 nm	123 nm
h_{17}	217.6 nm	231 nm
u_y	356 nm	359 nm
fillet radius	25 nm	25 nm
δ_x	4.2	3.68
M	2.55	2.55

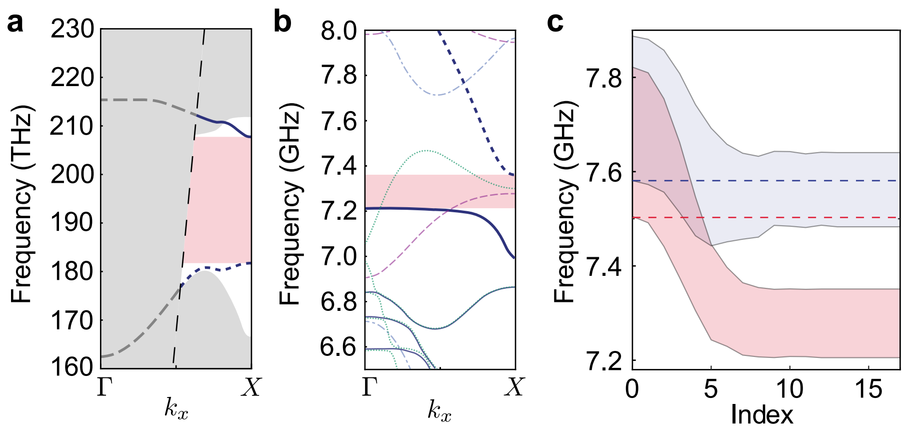

Supplementary Figure 2. **Effective waveguide band structure.** FEM simulations of the waveguide unit cell (see Fig. 1a of the main text) of device B displaying its **a**, photonic and **b**, acoustic band structures. Solid dark blue bands represent modes with symmetry groups where $\sigma_z = \sigma_y = 1$, meaning they are symmetric for reflections across the xy and zx-planes. Dotted green curves are $\sigma_z = -\sigma_y = 1$, dashed purple curves are $\sigma_z = \sigma_y = -1$, and light blue dash-dotted curves denote $-\sigma_z = \sigma_y = 1$. Dark blue curves indicate the bands giving rise to the confined optical and acoustic modes. Results for device A are qualitatively similar. The grey shaded areas correspond to the continuum of propagating modes outside of the boomerang bandgap. The dashed grey curves denote the leaky modes above the light-line (dashed black line). **c**, Mechanical bandgap as a function of cell index. The blue (red) shaded region shows bandgaps for device A (B).

Supplementary Note 3. GROUND STATE COOLING ANALYSIS

The impact on the phonon occupancy if the additional modes appearing at high input powers are neglected (or not) is shown in the Fig. Supplementary Figure 3. A single-mode fit overestimates the area of the mechanical mode of interest and provides an upper bound for the phonon occupancy of $n_\mathrm{m} \approx 0.5$. We find that the single-mode fit also yields mechanical linewidths appreciably larger than the linear trend expected within the optomechanical description, i.e. $\gamma_\mathrm{m} = (1 + C)\gamma_\mathrm{m}^0$, indicating that a larger g_0 would be required to explain our experiments if the satellite modes were not included in the fit.

Lastly, we estimate the optomechanical coupling rate of the spurious mode with strongest signal, as shown in Fig. Supplementary Figure 3. From backaction cooling (linewidth broadening) we estimate a $g_0/2\pi \approx 55(10)\,\mathrm{kHz}$, which is over an order of magnitude lower than the breathing mode's.

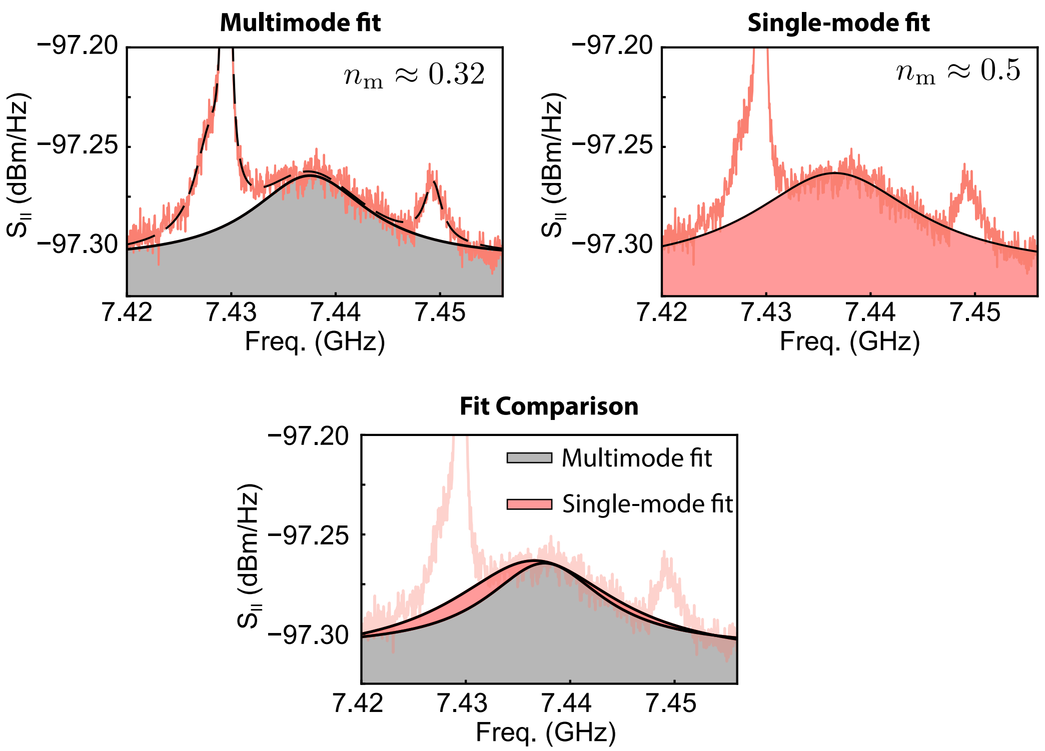

Supplementary Figure 3. **Different models for ground state cooling data.** Comparison of multimode and single-mode fit for the mechanical spectrum during red sideband pumping. We find that using a single-mode model leads to overestimating both the thermal occupancy and linewidth broadening of the mechanical breathing mode.

Supplementary Note 4. ONSET OF THE STRONG COUPLING REGIME

Additional optomechanically-induced transparency (OMIT) experiments were performed below and above the threshold for the strong coupling regime, as exemplified in Supplementary Figure 4a and b. The splitting resulting from OMIT – a direct measurement of g/π – agrees well with predictions assuming the independently measured g_0 from cooling and heating experiments described in the main text, as displayed in Supplementary Figure 4c.

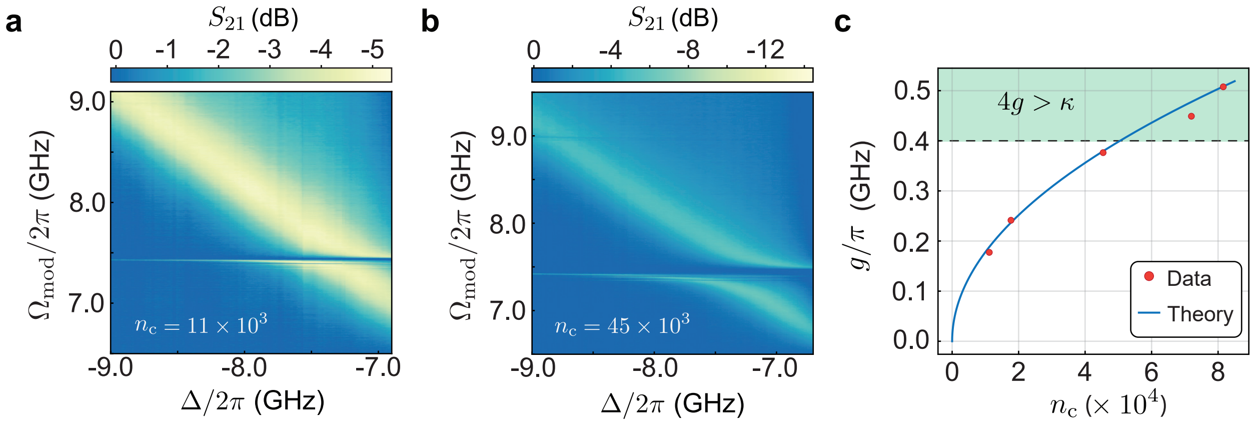

Supplementary Figure 4. **Onset of the strong coupling regime.** Optomechanically-induced transparency measurement as a function of laser-cavity detuning Δ for **a**, $n_c = 11 \times 10^3$ and **b**, $n_c = 45 \times 10^3$. **c**, Mode splitting due to OMIT as a function of n_c. The green-shaded area denotes the optomechanical strong coupling regime. The theory trace was obtained using the measured g_0 from dynamical backaction experiments shown in the main text.

Supplementary Note 5. OPTICAL STABILITY AT 3 K

We park the pump laser at the red mechanical sideband ($\Delta = -\omega_\mathrm{m}$) of the cavity and modulate the incoming light using an electro-optical intensity modulator using a vector network analyzer. By sweeping the modulation frequency Ω_mod and recording the beat-signal of the reflected signal on a high-speed photodiode, we extract the detuning of our laser with respect to the optical mode. Supplementary Figure 5 shows the change in detuning ($\Delta(n_\mathrm{c}) + \omega_m$) between the optical mode and the free-running laser dependent on the cavity photon occupation. We observe shifts $< 150\,\mathrm{MHz}$ even for cavity occupations up to $n_\mathrm{c} \approx 5000$.

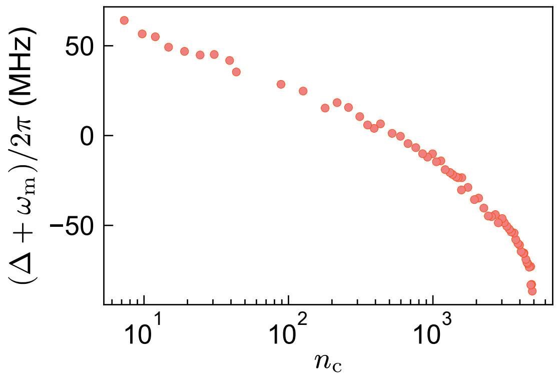

Supplementary Figure 5. **Optical Power stability.** Frequency shift of the optical mode versus cavity photon occupation n_c measured using coherent spectroscopy of the optical mode.

Supplementary Note 6. MEASUREMENT SETUP

A schematic of the full experimental setup used in the sideband asymmetry, ground state cooling, and optomechanical strong coupling is provided in Supplementary Figure 6. Two tunable diode lasers (PurePhotonics PPCL300) are first intensity-stabilized with electro-optic modulators (EOMs), and then frequency-stabilized using temperature-stabilized fiber Fabry-Pérot filters (F1 and F2). A fast wavelength-scanning laser (Freedom Photonics FP4209) assists with fiber-to-chip coupling in the dilution refrigerator using a lensed fiber mounted on cryogenic positioners (Attocube). Two acousto-optic modulators (AOMs) in-series are simultaneously pulsed, utilizing a Quantum Machine OPX, to generate the optical pump pulse with a high on-off ratio ($> 90\,\mathrm{dB}$) for the pulsed operation. On the other hand, sending a constant signal to the AOMs enables continuous-wave (CW) operation. A pair of narrow-band Fabry-Pérot cavities (FA and FB), with bandwidth $\approx 15\,\mathrm{MHz}$, suppress the pump photons with a joint suppression of $> 90\,\mathrm{dB}$ with respect to the sideband photons. Multiple MEMS optical switches route light to various segments of the setup: an EOM to generate and sweep sidebands (used for OMIT measurements, locking filter cavities FA and FB, calibrating the cooling measurement), an erbium-doped fiber amplifier (EDFA) to boost input power to the device (for the strong-coupling measurement), filter cavities FA and FB to suppress the pump before single photon detection (Photonspot, used for pulsed asymmetry measurements), and a high-speed photodetector (for OMIT, cooling, and strong-coupling measurements).

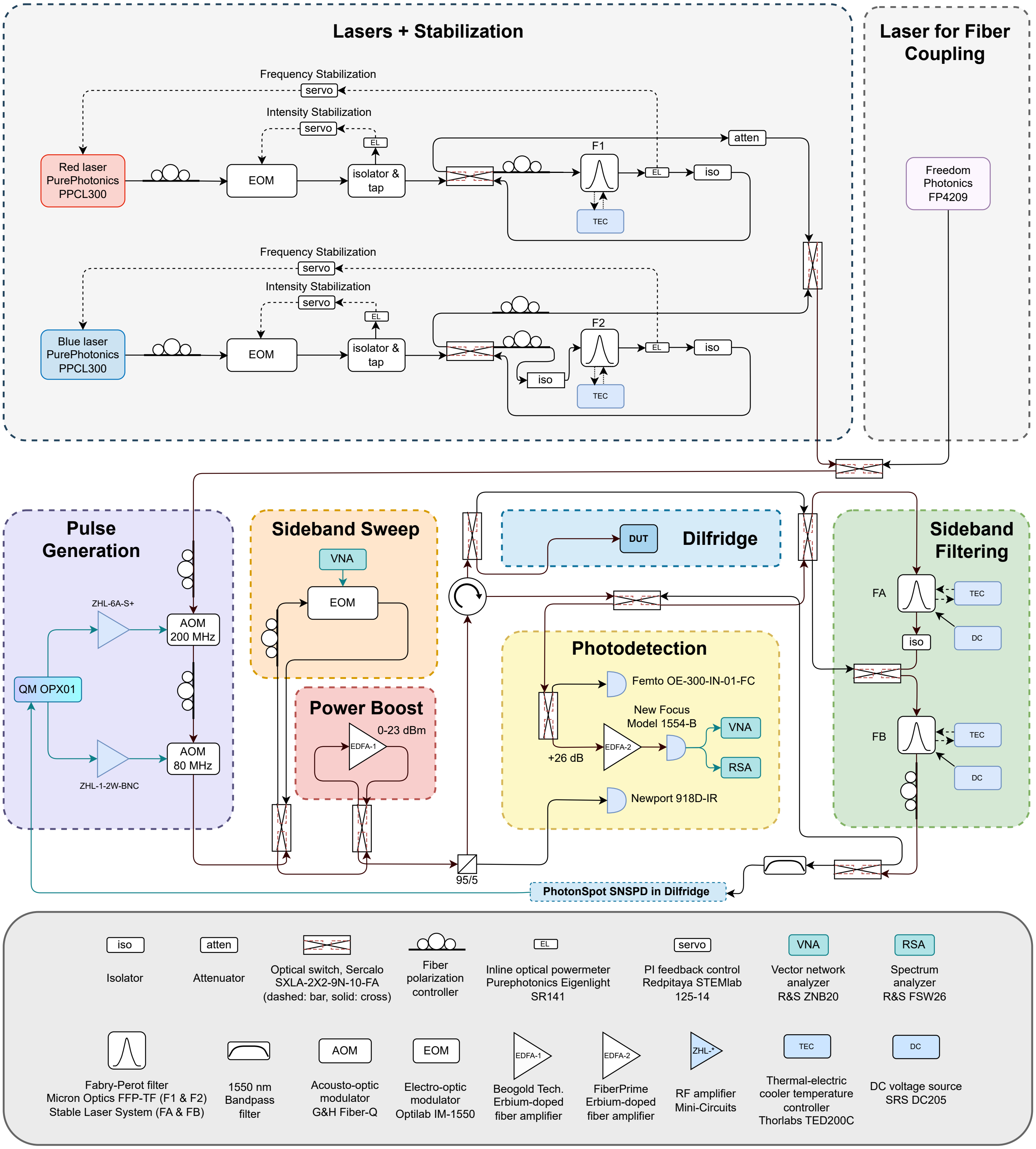

Supplementary Figure 6. **Measurement Setup.** Diagram representing the full optical setup used in the pulsed and CW measurements.

[1] Safavi-Naeini, A. H. & Painter, O. Design of optomechanical cavities and waveguides on a simultaneous bandgap phononic-photonic crystal slab. *Optics Express* **18**, 14926–14943 (2010).
[2] Ren, H. *et al.* Two-dimensional optomechanical crystal cavity with high quantum cooperativity. *Nature Communications* **11**, 3373 (2020).
[3] Chiappina, P. *et al.* Design of an ultra-low mode volume piezo-optomechanical quantum transducer. *Optics Express* **31**, 22914 (2023).
[4] Aram, M. H. & Khorasani, S. Optomechanical coupling strength in various triangular phoxonic crystal slab cavities. *Journal of the Optical Society of America B* **35**, 1390 (2018).
